# Skeletal Muscle Deconditioning in Breast Cancer Patients Undergoing Chemotherapy: Current Knowledge and Insights From Other Cancers

**DOI:** 10.3389/fcell.2021.719643

**Published:** 2021-09-14

**Authors:** Joris Mallard, Elyse Hucteau, Thomas J. Hureau, Allan F. Pagano

**Affiliations:** ^1^Institut de Cancérologie Strasbourg Europe (ICANS), Strasbourg, France; ^2^Centre de Recherche en Biomédecine de Strasbourg (CRBS), Fédération de Médecine Translationnelle, UR 3072, Université de Strasbourg, Strasbourg, France; ^3^Faculté des Sciences du Sport, Centre Européen d’Enseignement de Recherche et d’Innovation en Physiologie de l’Exercice (CEERIPE), Université de Strasbourg, Strasbourg, France

**Keywords:** cancer cachexia, muscle atrophy, protein turnover, intermuscular adipose tissue, inflammatory cytokines, mitochondria, oxidative stress, satellite cells

## Abstract

Breast cancer represents the most commonly diagnosed cancer while neoadjuvant and adjuvant chemotherapies are extensively used in order to reduce tumor development and improve disease-free survival. However, chemotherapy also leads to severe off-target side-effects resulting, together with the tumor itself, in major skeletal muscle deconditioning. This review first focuses on recent advances in both macroscopic changes and cellular mechanisms implicated in skeletal muscle deconditioning of breast cancer patients, particularly as a consequence of the chemotherapy treatment. To date, only six clinical studies used muscle biopsies in breast cancer patients and highlighted several important aspects of muscle deconditioning such as a decrease in muscle fibers cross-sectional area, a dysregulation of protein turnover balance and mitochondrial alterations. However, in comparison with the knowledge accumulated through decades of intensive research with many different animal and human models of muscle atrophy, more studies are necessary to obtain a comprehensive understanding of the cellular processes implicated in breast cancer-mediated muscle deconditioning. This understanding is indeed essential to ultimately lead to the implementation of efficient preventive strategies such as exercise, nutrition or pharmacological treatments. We therefore also discuss potential mechanisms implicated in muscle deconditioning by drawing a parallel with other cancer cachexia models of muscle wasting, both at the pre-clinical and clinical levels.

## Introduction

Cancer represents the leading cause of death worldwide and a substantial barrier to increasing life expectancy. Among the different cancer sites, breast cancer is the most commonly diagnosed cancer, with 11.7% of total cases and 6.9% of cancer deaths (Sung et al., 2021). Effective therapy of breast cancer requires a multidisciplinary approach including surgery, radiotherapy, neoadjuvant and/or adjuvant therapies. Currently, neoadjuvant and adjuvant chemotherapies are extensively used in breast cancer patients to reduce tumor development and improve disease-free survival, but also leads to severe off-target side-effects (Maughan et al., 2010; Redden and Fuhrman, 2013; Fisusi and Akala, 2019; Schirrmacher, 2019). Among these treatment-related side effects, both pre-clinical and clinical studies highlighted that chemotherapeutic agents result in major skeletal muscle deconditioning and, together with exacerbated fatigue, are part of a vicious cycle which negatively impacts their quality of life (Berger et al., 2015; Caan et al., 2018; Aleixo et al., 2019; Cespedes Feliciano et al., 2019; Hiensch et al., 2019; Mallard et al., 2020). Although breast cancer represents the most deadly female cancer, 5-year survival rate is over 90% (National Cancer Institute, Surveillance, Epidemiology, and End Result program, 2019) emphasizing the critical need to fight long-lasting effects observed in survivors such as skeletal muscle deconditioning.

Skeletal muscle deconditioning is a direct consequence of global muscle homeostasis perturbation, leading to both structural and functional alterations that will translate into a decrease in muscle mass and/or force as well as an increase in fatigability (Chopard et al., 2009; Baldwin et al., 2013; Brioche et al., 2016; Cruz-Jentoft et al., 2019; Arc-Chagnaud et al., 2020). In the context of cancer patients, skeletal muscle atrophy represents a major characteristic of cachexia, which can be defined as an ongoing loss of skeletal muscle mass that cannot be fully reversed with nutrition and leading to functional alterations (Fearon et al., 2011). It is now well admitted that cancer cachexia is one of the most life-threatening aspects of cancer. Indeed, it has been shown that cachexia substantially increases sedentary behavior, functional impairment, loss of autonomy, quality of life degradation, surgical risks and overall adverse effects of chemotherapy (Fouladiun et al., 2007; Fearon et al., 2011; Roberts et al., 2013; Wallengren et al., 2013; Mason et al., 2016; Rutten et al., 2016; Schwarz et al., 2017; Baracos et al., 2018; Daly et al., 2018). Importantly, cachexia is also strongly correlated with a decrease in cancer patients survival and is actually the leading cause of death in cancer (Warren, 1932; Martin et al., 2015; Deluche et al., 2018; Huh et al., 2020). Thus, the management of skeletal muscle deconditioning during cancer and its treatment represents a major challenge for healthcare, particularly in breast cancer patients, considering both the high incidence of new cases (Sung et al., 2021) and the prevalence of cancer cachexia (∼25%) in breast cancer patients (Baracos et al., 2018). Even if, compared to other cancers, breast cancer does not display the highest prevalence of cachexia, it is important to note that cachexia diagnosis is based on global weight loss (Fearon et al., 2011), and not only muscle mass loss, which likely led to an underestimation of cachexia prevalence in clinical practice (Roeland et al., 2017).

To date, the cellular mechanisms of skeletal muscle deconditioning are of great importance and have been extensively reviewed in healthy people, elderly as well as in relation with many chronic diseases (Sandri, 2008; Chopard et al., 2009; Bodine, 2013; Bonaldo and Sandri, 2013; Schiaffino et al., 2013; Argilés et al., 2014; Bowen et al., 2015; Brioche et al., 2016; Petruzzelli and Wagner, 2016; Baracos et al., 2018; Larsson et al., 2019; Dolly et al., 2020; Silva et al., 2020; Vainshtein and Sandri, 2020; Sartori et al., 2021). However, in comparison with the knowledge accumulated through decades of intensive research with many different animal and human models, a comprehensive understanding of the cellular processes implicated in breast cancer-mediated muscle deconditioning is still needed in order to develop efficient strategies to counteract it.

This review focuses on recent advances in both macroscopic changes and cellular mechanisms implicated in skeletal muscle deconditioning of breast cancer patients, specifically as a consequence of chemotherapy treatment. This review also aims to highlight other potential mechanisms by drawing a parallel with cancer cachexia models of muscle wasting, both at the pre-clinical and clinical levels.

## Chemotherapy-Induced Skeletal Muscle Macroscopic Alterations in Breast Cancer Patients

Two families of chemotherapeutic agents are commonly used in clinical practice for breast cancer patients: anthracyclines (i.e., doxorubicin or epirubicin) leading to DNA damage, and taxanes (i.e., docetaxel or paclitaxel) acting as cytoskeletal disruptors (Shah and Gradishar, 2018; Willson et al., 2019). Importantly, non-hormone-dependent (i.e., triple-negative or HER2-positive) breast cancer treatment also includes immunotherapy, a promising new field in breast cancer therapy (Emens, 2018; Keenan and Tolaney, 2020). If immunotherapy has been identified to induce severe cardiotoxicity (Behr et al., 2001; Rochette et al., 2015; Bregni et al., 2016; Varricchi et al., 2018), there is no study to date with a focus on skeletal muscle. On the other hand, chemotherapeutic agents are recognized to contribute to skeletal muscle deconditioning, resulting in an altered quality of life, increased treatment-related toxicity, and to an increased mortality risk (Rier et al., 2016; Shachar et al., 2017; Deluche et al., 2018; Trestini et al., 2018; Cespedes Feliciano et al., 2019; Huh et al., 2020). To date, several skeletal muscle structural and functional alterations were identified (loss of muscle mass and force, altered quality) with severe consequences on exercise tolerance.

### Muscle Mass

Although it is widely accepted that chemotherapy induces skeletal muscle loss in breast cancer patients, very few studies clearly demonstrated it. Indeed, by excluding all non-longitudinal studies (i.e., with no pre *vs*. post-chemotherapy assessments) and lean body mass measurements (i.e., with no assessment of muscle mass in isolation), only two studies emerged (Rossi et al., 2020; Wiederin et al., 2020). Both studies demonstrated a decrease in pectoralis muscle area after chemotherapy. Wiederin et al. (2020) found a 10% reduction in muscle mass using magnetic resonance imaging in a cohort of breast cancer (*N* = 221), sarcoma (*N* = 115) and lymphoma (*N* = 216) female patients. In breast cancer only, Rossi et al. (2020) found a 15% reduction in muscle mass by using CT Scan. Surprisingly, we were unable to find any other longitudinal study on whole-body or locomotor muscle mass for breast-cancer patients undergoing chemotherapy. As a loss of skeletal muscle mass is strongly associated with poor functional outcomes (Fearon et al., 2011; Baracos et al., 2018; Cruz-Jentoft et al., 2019; Aleixo et al., 2020a) and chemotherapy efficacy (Caan et al., 2018; Lee et al., 2021) in breast cancer patients, further studies are needed to better characterize the loss of muscle mass in order to counteract it effectively thereafter.

### Muscle Force

On the other hand, the impact of chemotherapy treatment on muscle force is more documented. Numerous studies, with various protocols of force evaluation (handgrip, isometric knee extension, mid-thigh pull, and shoulder strength, etc.), found inconsistent results on chemotherapy-treated breast cancer patients. Indeed, some longitudinal studies (Schmidt et al., 2015; Ramos da Silva et al., 2021) documented no change in isometric muscle force in both lower limbs (quadriceps femoris muscle) and upper limbs (latissimus dorsi, pectoralis, and handgrip muscles), while others found a significant reduction from −4 to −17% in handgrip or knee extensors muscle force (van Waart et al., 2015; Gadéa et al., 2018; Mijwel et al., 2018a; CeŠeiko et al., 2020; Toth et al., 2020). Discrepancies in study protocols (study duration, measurements timepoints, and treatments administered) and in the methods of force evaluation (isometric *vs.* isokinetic contractions, different muscle groups investigated) may explain these contrasting results. Other studies also highlighted a decrease in muscle force of breast cancer patients undergoing chemotherapy in comparison with healthy women (Klassen et al., 2017; Marques et al., 2020), supporting the fact that chemotherapeutic agents may affect skeletal muscle force production.

### Muscle Quality

There is a growing body of evidence that the loss of muscle strength and power mostly exceeds the loss of muscle mass observed in many diseases or inactivity experiments, emphasizing that a deterioration in muscle quality could explain the loss in force and lead to functional impairments (di Prampero and Narici, 2003; Brioche et al., 2016; Pagano et al., 2018; CeŠeiko et al., 2020; Toth et al., 2020). Muscle quality can be assessed through different techniques, including magnetic resonance imaging, computed tomography or ultrasound echography (Karampinos et al., 2012; Addison et al., 2014; Aubrey et al., 2014; Khan et al., 2019; Stock and Thompson, 2021), that allows the detection and quantification of abnormalities in skeletal muscle composition. Among these abnormalities, intermuscular adipose tissue (IMAT) accumulation is particularly of interest. Indeed, these muscle fatty infiltrations (i.e., adipocytes located between muscle fibers and muscle groups), also referred as myosteatosis, are known to be associated with inactivity (Manini et al., 2007; Leskinen et al., 2009; Tuttle et al., 2011; Pagano et al., 2018), pathologies (Gorgey and Dudley, 2007; Wren et al., 2008; Karampinos et al., 2012; Gallagher et al., 2014; Uezumi et al., 2014b) and have been particularly investigated in sarcopenia (Goodpaster et al., 2000, 2001; Song et al., 2004; Marcus et al., 2010; Brioche et al., 2016). An accumulation of IMAT is closely linked to poor muscle quality and therefore muscle dysfunction (Jubrias et al., 1997; Visser et al., 2002, 2005; Delmonico et al., 2009; Marcus et al., 2010; Murphy et al., 2011; Tuttle et al., 2011; Beavers et al., 2013). In the specific context of cachexia, a reduction in muscle quality has been observed in breast cancer patients treated with chemotherapeutic agents. In a longitudinal study, metastatic breast cancer patients showed an altered muscle attenuation after taxane-based chemotherapy, indicating a decrease in muscle quality (Rier et al., 2018). In a cross-sectional study, breast cancer survivors who received anthracyclines were compared to control subjects and a clear increase in thigh IMAT content (∼30%) have been found and was interestingly correlated with an impaired cardiorespiratory fitness (Beaudry et al., 2020). Another cross sectional study highlighted an increased IMAT content in cancer patients (including breast-cancer patients) when compared to non-cancer individuals (Reding et al., 2019) and also showed a good correlation with the development of exercise intolerance.

### Exercise Tolerance

As a consequence of the abovementioned skeletal muscle alterations, combined with a well-known cardiotoxicity (Bird and Swain, 2008; Kazemi-Bajestani et al., 2014; Nicolazzi et al., 2018; Varricchi et al., 2018; Jerusalem et al., 2019), chemotherapy is strongly impacting exercise tolerance. In clinical setting, the six-minute walk test (6MWT) represents a reference test reflecting exercise tolerance and is widely used in various pathologic populations (Enright, 2003; Agarwala and Salzman, 2020), including cancer patients (Galiano-Castillo et al., 2016; Wesolowski et al., 2020). A recent systematic-review reported, through the analysis of 21 original studies using the 6MWT, that 1,084 breast cancer patients (including both patients under treatment and survivors) showed a 24% reduction in performance compared to 878 healthy people (But-Hadzic et al., 2021). Aside the 6MWT, widely used as an indirect measurement of cardiorespiratory fitness, the assessment of the maximal oxygen consumption (V̇O_2*max*_) represents the gold standard measurement of exercise tolerance (Astrand and Saltin, 1961; Schumacher et al., 2019). Interestingly, consistent results between the 6MWT and V̇O_2*max*_ were found in breast cancer patients. Indeed, another systematic review reported, from the analysis of 27 clinical trials, a significant 25% reduction in V̇O_2*max*_ after chemotherapy treatment compared to healthy sedentary women (Peel et al., 2014). This cardiorespiratory deconditioning seems to strengthen the development of cancer-related fatigue and particularly physical fatigue (Neil et al., 2013), with consequences on exercise intolerance. Indeed, physical fatigue, assessed by the reduction in force during the repetition of maximal voluntary contractions, has been found to be exacerbated in breast cancer patients undergoing chemotherapy treatment compared to healthy individuals (Klassen et al., 2017), negatively impacting their exercise tolerance. Together with the decrease of skeletal muscle mass, a reduction in exercise capacity is also strongly associated with higher risk of adverse outcomes such as treatment-induced toxicity, mortality or functional impairment (Jones et al., 2012; Peel et al., 2014; Foulkes et al., 2019; Yu et al., 2020).

## Cellular Mechanisms of Skeletal Muscle Deconditioning in Breast Cancer Patients: What Do We Know?

**FIGURE 1:**
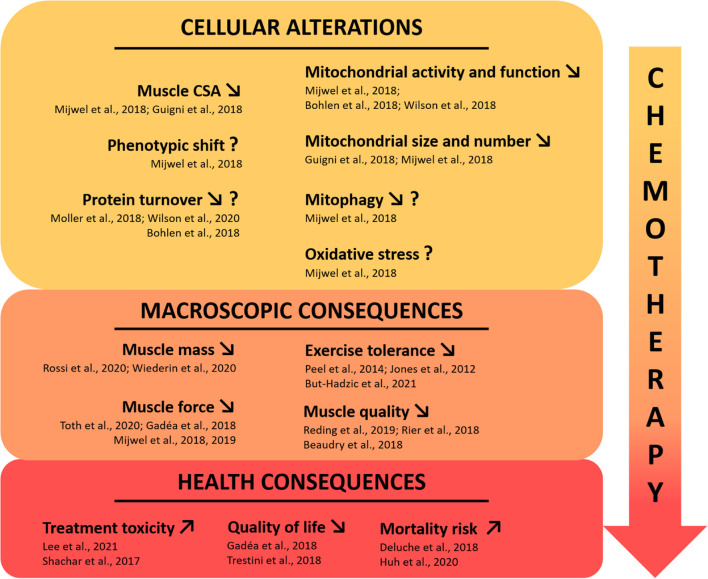
Current state of knowledge on skeletal muscle deconditioning in breast cancer patients. The use of chemotherapeutic agents in clinical breast cancer treatment is directly affecting skeletal muscle tissue, leading to major muscle deconditioning. To date, few clinical studies investigated the underlying cellular mechanisms that may be responsible for the macroscopic changes highlighted in breast cancer patients, affecting muscle function and having severe health consequences. The question mark indicates inconsistent results or a mechanism that needs to be consolidated with more studies. CSA, cross sectional area.

Skeletal muscle biopsy (e.g., using Bergström needle) is the only technique allowing full investigation of the cellular mechanisms of muscle deconditioning (Bergstrom, 1975; Tarnopolsky et al., 2011). To date, only six clinical studies, published in seven different publications, used muscle biopsies in early breast cancer patients (stage I–III) to decipher mechanisms of muscle deconditioning (Lønbro et al., 2017; Bohlen et al., 2018; Guigni et al., 2018; Mijwel et al., 2018b; Møller et al., 2019; Toth et al., 2020; Wilson et al., 2020). Altogether, these studies highlighted several important aspects of muscle deconditioning detailed below and outlined in Figure 1.

### Decrease in Skeletal Muscle Fibers Cross-Sectional Area and Phenotypic Shift

Muscle fibers CSA is one of the most substantial measurement of muscle deconditioning at the cellular level. Recently, Mijwel et al. (2018b) and Guigni et al. (2018) showed a clear decrease in both type I and type II vastus lateralis muscle fibers CSA after anthracycline-cyclophosphamide and taxane-based chemotherapy treatment. Interestingly, Mijwel et al. (2018b) demonstrated it through a longitudinal study while Guigni et al. (2018) have done it with a cross-sectional study design, by comparing breast cancer patients to healthy individuals. To highlight the substantial magnitude of the decrease in overall muscle fibers CSA during chemotherapy in breast cancer patients, it should be noted that this decrease was comparable to the considerable effect of 60 years of healthy aging (Lexell et al., 1988). If comparing muscle CSA of breast cancer patients under chemotherapy for 4 months with 60 years of aging is insightful to emphasize the profound impact of chemotherapy on the skeletal muscle apparatus, it is not fully accurate as other mechanisms are involved and interact with CSA differently in cancer *vs.* aging. Two other longitudinal studies found no reduction in muscle fibers vastus lateralis CSA during chemotherapy including taxanes, cyclophosphamide, doxorubicin, and carboplatin (Lønbro et al., 2017; Toth et al., 2020). However, in these studies, the second muscle biopsy was performed after ∼5 weeks and might explain the lack of atrophy as the effects of chemotherapeutic agents on skeletal muscle are strongly suggested to be cumulative. It is important to note that *in vitro* and *in vivo* studies also demonstrated the negative impact of both chemotherapeutic agents (McLoon et al., 1998; Gouspillou et al., 2015; Min et al., 2015; Barreto et al., 2016; Guigni et al., 2018) and breast cancer-bearing mice models (Hesse et al., 2019; Wang et al., 2021) on skeletal muscle structure, strengthening the results obtained in clinical studies.

Concerning fiber type distribution, only Mijwel et al. (2018b) reported significant changes, with a reduced proportion of type I muscle fibers after chemotherapy treatment. This potential type I to type II phenotypic shift is classically found in muscle disuse models (Baldwin et al., 2013) while the opposite is observed with aging (Larsson et al., 2019). This suggests that muscle deconditioning in breast cancer patients might also be driven by a decrease in overall physical activity during their treatment (De Groef et al., 2018; Gadéa et al., 2018; Yildiz Kabak et al., 2020), a well-known trigger of protein turnover dysregulation.

### Protein Turnover

If a large number of excellent reviews have already documented the critical role of protein turnover homeostasis in the mechanisms related to skeletal muscle atrophy (Sandri, 2008; Chopard et al., 2009; Bodine, 2013; Bonaldo and Sandri, 2013; Schiaffino et al., 2013; Brioche et al., 2016; Larsson et al., 2019; Vainshtein and Sandri, 2020; Sartori et al., 2021) including in cancer cachexia (Argilés et al., 2014; Bowen et al., 2015; Petruzzelli and Wagner, 2016; Baracos et al., 2018; Dolly et al., 2020; Silva et al., 2020), little is known in the unique context of breast cancer. Indeed, only four studies investigated the mechanisms related to protein turnover homeostasis in breast cancer patients (Bohlen et al., 2018; Mijwel et al., 2018b; Møller et al., 2019; Wilson et al., 2020). Two publications from the same research team showed, through RNAseq analysis on pectoralis muscle, an increased expression of genes related to ubiquitin-mediated proteolysis and a decreased expression of genes related to ribosomes (Bohlen et al., 2018; Wilson et al., 2020). These results potentially indicate an altered protein turnover balance, with a reduced protein synthesis and an increased protein breakdown. Mijwel et al. (2018b) did not find any changes in MuRF1 protein expression (a key E3 ligase implicated in the ubiquitin-proteasome system) after chemotherapy in breast cancer patients, nor concerning the autophagy pathway, with no changes in the protein expression of different key markers implicated in this pathway (i.e., p-Ulk1, LC3B-II/I ratio, beclin-1, all reflecting autophagosome formation). These results could be explained by the “late” time-point of biopsy collection in this study as cellular processes triggering muscle atrophy, particularly those related to protein breakdown, tend to go back to “normal” expression profiles when the muscle atrophy is well established (Ferreira et al., 2008; Hanson et al., 2013; Atherton et al., 2016; Kawanishi et al., 2018). Finally, the study conducted by Møller et al. (2019) also investigated proteins involved in signaling pathways implicated in protein turnover from vastus lateralis muscle. Very surprisingly, they found a decreased protein expression of the E3 Ligases MAFbx and MuRF1 as well as an increase in p62 and phosphorylated-Ulk1 expression (Ser757), suggesting a decreased activity of the ubiquitin proteasome and autophagy systems, respectively. However, it is important to highlight that 9 out of 10 patients included in this study performed the baseline biopsy after at least one cycle of chemotherapy with epirubicin and doxorubicin (Lønbro et al., 2017). Given the aggressiveness of chemotherapy treatments, this is a serious methodological bias that likely altered “baseline” measures, and therefore, conclusions. Another limitation lays in the heterogeneous population of cancer patients investigated (i.e., seven patients with breast-cancer, one patient with head and neck cancer, one patient with rectal cancer, and one patient with sarcoma). To sum up, there are strong discrepancies between studies that investigated pathways of protein synthesis and breakdown in breast cancer patients undergoing chemotherapy. Further studies are needed as the understanding of these processes is critical to counteract the skeletal muscle atrophy outlined above.

### Mitochondrial Alterations

Mitochondrial alterations represent, to date, one of the most investigated aspect of muscle deconditioning in breast cancer, especially in response to chemotherapeutic agents. In clinical studies, the RNAseq analysis used by both Bohlen et al. (2018) and Wilson et al. (2020) showed a clear dysregulation of genes implicated in mitochondrial function and oxidative phosphorylation. Interestingly, the authors showed a decrease in multiple genes implicated in the electron transport chain, antioxidant capacity, and altered PPAR signaling (including PGC-1α), emphasizing that mitochondria and overall energy homeostasis may be perturbed in breast cancer patients treated with chemotherapeutic agents. Guigni et al. (2018) confirmed a clear decrease in mitochondrial content and size for breast cancer patients compared to healthy matched controls, in both the intermyofibrillar and subsarcolemmal compartments. The authors concluded that these alterations, due to the mitotoxic effects of antineoplastic drugs, may constitute a possible explanation to the high prevalence of exercise intolerance and fatigue in all cancer’s types, including those not typically prone to cachexia such as breast cancer patients. Finally, the longitudinal study of Mijwel et al. (2018b) highlighted a decrease in citrate synthase activity with chemotherapy. The decrease in citrate synthase activity, a marker for mitochondrial quantity (Larsen et al., 2012), is in line with the results of Guigni et al. (2018) and confirms the likely lower mitochondria quantity in breast cancer patients. This study also reports a decreased protein expression of PINK1, an essential protein implicated in the final stages of mitophagy, therefore suggesting a lower mitophagy process in breast cancer patients. In addition, no variation in protein levels of Parkin has been detected in this study, nor those of the autophagy pathway, clearly indicating that mitophagy is not upregulated and that future studies should investigate this mitochondrial quality control pathway. Finally, an increased protein expression of SOD2, an essential antioxidant enzyme and redox signaling trigger through H_2_O_2_ production (Zou et al., 2017), was also found. Alone, this result does not permit to raise any conclusion whether it reflects an increase in antioxidant defenses or, at the opposite, a compensation for an increase in oxidative stress (i.e., superoxide anion) linked to the chemotherapeutic treatment. Clearly, future studies with protein expression analysis of oxidative stress and antioxidant pathways as well as enzymes activities are still necessary to understand the potential implication of redox balance in skeletal muscle deconditioning of breast cancer patients.

## Potential Other Cellular Mechanisms of Muscle Deconditioning in Breast Cancer Patients: What Can We Learn From Other Cancers?

**FIGURE 2:**
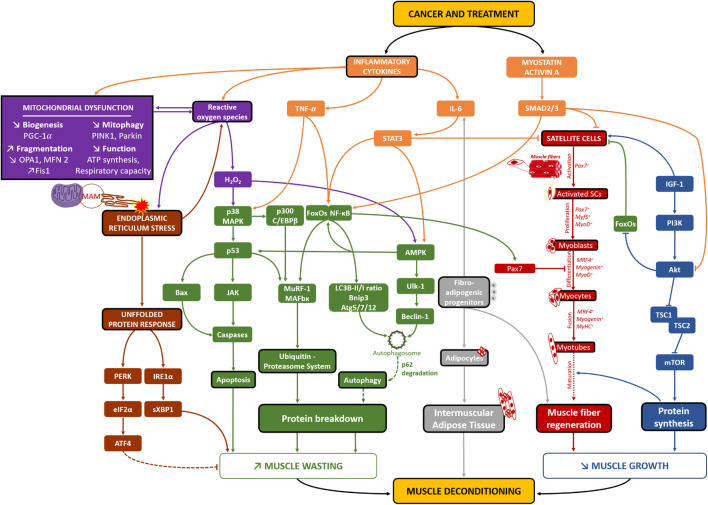
Potential cellular mechanisms of skeletal muscle deconditioning in breast cancer patients. Through the analysis of clinical and pre-clinical studies related to all cancer’s types, several different pathways may be implicated in skeletal muscle deconditioning in breast cancer patients. Both the cancer *per se* and its treatment lead to increased levels of inflammatory cytokines and Myostatin/Activin A pathways (in orange), consequently decreasing protein synthesis (in blue) and increasing pathways implicated in protein breakdown (in green). The activation of the autophagy-lysosomal system needs to be elucidated as an increase in autophagosomes formation has been consistently found as well as increased levels of p62, suggesting that lysosome activity might be disrupted in cancer cachexia, leading to no change in autophagy flux (green dotted line). The combination of high levels of inflammatory cytokines, ROS and mitochondrial altered dynamics, biogenesis and function (in purple) would also lead to increased protein breakdown and apoptosis. On the other hand, it would also lead to high levels of endoplasmic reticulum stress, resulting in an increase in the UPR system (in brown) and consequently protein breakdown. The involvement of the PERK/eiF2α/ATF4 pathway, known to be implicated in non-cancer models of muscle atrophy, needs to be clarified, as it might surprisingly be necessary to counteract muscle atrophy in cancer-related models of muscle atrophy (brown dotted line). Moreover, different studies also highlighted a reduction in the number of satellite cells (SCs), their capacity to differentiate and skeletal muscle capacity to regenerate, emphasizing that cancer-related muscle atrophy may also result from muscle altered repair/regrowth (in red). Finally, increased presence of fibro-adipogenic progenitors might also be implicated in muscle deconditioning as they are implicated in both IL-6-mediated muscle atrophy (in non-cancer models of muscle atrophy) and Intermuscular adipose tissue development (in gray). Altogether, these cellular mechanisms might play an important role in breast cancer-related skeletal muscle deconditioning and clearly need to be further investigated through clinical studies using muscle biopsies. MAM, mitochondria-associated ER membranes.

Based on the knowledge accumulated through decades of intensive research, this part of the review aims to identify potential cellular mechanisms responsible for skeletal muscle deconditioning in breast cancer patients by drawing a parallel with pre-clinical studies and other cancers models of muscle wasting. As summarized in Figure 2, we have limited our review to the main and well admitted mechanisms of muscle wasting in cancer; our list is therefore not exhaustive. Among the large variety of studies discussed hereafter, we found few studies related to skeletal muscle plasticity conducted on mouse models of breast cancer while several pre-clinical studies explored the effect of doxorubicin administration, one of the most commonly used chemotherapeutic agents to treat breast cancer patients. This lack of specific investigations indicates a major imbalance in comparison with other cancers and also emphasizes the need to remain cautious with the mechanisms identified thereafter as they mainly stem from the analysis of different cancers and treatments. However, it will provide future directions for researchers willing to investigate specifically the mechanisms of muscle deconditioning in breast cancer.

### Protein Turnover

The sensitive balance between protein synthesis and protein breakdown is the major mechanism regulating muscle mass (Chopard et al., 2009; Schiaffino et al., 2013; Bowen et al., 2015; Argilés et al., 2019; Silva et al., 2020; Vainshtein and Sandri, 2020).

Skeletal muscle protein synthesis is mainly promoted by the PI3K-Akt-mTOR pathway and cachexia patients with pancreatic carcinoma or lung cancer demonstrated an altered PI3K-Akt-mTOR signaling (Schmitt et al., 2007; Murton et al., 2017), a result also found in various pre-clinical models (White et al., 2011; Padrão et al., 2013; Gallot et al., 2014; Puppa et al., 2014; Bohnert et al., 2016; Chen M. C. et al., 2016; de Lima Junior et al., 2016; Sun et al., 2016; Chacon-Cabrera et al., 2017; Quan-Jun et al., 2017; Nissinen et al., 2018; Salazar-Degracia et al., 2018). Importantly, both clinical (Bennegård et al., 1984; Emery et al., 1984; Dworzak et al., 1998) and pre-clinical studies (Beck et al., 1991; Smith and Tisdale, 1993; Samuels et al., 2001; Smith et al., 2004; Constantinou et al., 2011; Nissinen et al., 2016, 2018; Toledo et al., 2016; Antoun and Raynard, 2018; Cruz et al., 2019; Costamagna et al., 2020) highlighted a reduction in muscle protein synthesis, emphasizing that a reduction in protein synthesis may explain, at least in part, the muscle deconditioning occurring in cancer patients. Among all these studies, only two worked on rodents treated with doxorubicin and showed a reduced PI3K-Akt-mTOR signaling (de Lima Junior et al., 2016; Nissinen et al., 2016). Moreover, through RNAseq analysis, Wilson et al. (2019) also found an altered skeletal muscle mTOR signaling in breast cancer-bearing mice. These important studies clearly demonstrated that both breast cancer *per se* and the chemotherapeutic agents used in clinical setting to treat it may alter the main protein synthesis pathway in skeletal muscle, possibly leading to altered protein turnover.

On the other hand, protein breakdown includes two major pathways, the ubiquitin-proteasome and autophagy-lysosomal systems (UPS and autophagy, respectively), that are responsible for the degradation of most proteins and organelles in skeletal muscle cells. First, numerous pre-clinical studies observed an increase in skeletal muscle protein breakdown (Beck et al., 1991; Smith and Tisdale, 1993; Temparis et al., 1994; Baracos et al., 1995; Samuels et al., 2001; Smith et al., 2004; Silva et al., 2015; Toledo et al., 2016), demonstrating that a decrease in protein synthesis is not the only mechanism that could explain the loss muscle mass in cancer cachexia. UPS and autophagy pathways have been largely investigated both in clinical and pre-clinical studies. The UPS is almost unanimously found to be increased in cancer patients, particularly the “atrogenes” MAFbx and MuRF1 and the overall ubiquitination profile (Williams et al., 1999; Bossola et al., 2003; DeJong et al., 2005; Khal et al., 2005; Constantinou et al., 2011; Puig-Vilanova et al., 2015; Zhang et al., 2020). Importantly, the increase in UPS activity as well as mRNA/proteins implicated in this pathway is also consistently found in a large number of pre-clinical studies (Baracos et al., 1995; Gomes et al., 2001; Lecker et al., 2004; Acharyya et al., 2005; Khal et al., 2005; Moore-Carrasco et al., 2007; Zhou et al., 2010; Julienne et al., 2012; Padrão et al., 2013; Chacon-Cabrera et al., 2014, 2017; Gallot et al., 2014; Johnston et al., 2015; Silva et al., 2015; Bohnert et al., 2016; Chen M. C. et al., 2016; Hatakeyama et al., 2016; Sun et al., 2016; Toledo et al., 2016; Guo et al., 2017; Damrauer et al., 2018; Pin et al., 2018; Salazar-Degracia et al., 2018; Chen L. et al., 2019; Lee et al., 2019; Liu et al., 2019; Ranjbar et al., 2019; Bae et al., 2020; Huot et al., 2020), strengthening the fact that the UPS plays a major role in the protein breakdown aggravation. Concerning the autophagy system, clinical studies also demonstrated an increase in several important markers such as beclin1, Atg5, or LC3B-II/I ratio (Op den Kamp et al., 2012; Johns et al., 2014; Aversa et al., 2016; Pigna et al., 2016; de Castro et al., 2019; Zhang et al., 2020). Together with pre-clinical studies showing the same results (Penna et al., 2013, 2019a; Chacon-Cabrera et al., 2014; Bohnert et al., 2016; Salazar-Degracia et al., 2016, 2018; Sirago et al., 2017; Ballarò et al., 2019; Ranjbar et al., 2019), autophagy might also play a significant role in the increased protein breakdown of cancer patients. However, it appears of great importance to highlight that the majority of these studies also found an increase in p62 mRNA/protein expression, suggesting that if autophagosome formation is certainly increased, lysosome activity might be disrupted in cancer patients, leading to no modifications in autophagy flux (Penna et al., 2014; Klionsky et al., 2021). Furthermore, different studies showed that skeletal muscle protein breakdown is mostly ATP-dependent (i.e., UPS) in pre-clinical models of cancer cachexia (Temparis et al., 1994; Baracos et al., 1995; White et al., 2011), emphasizing again that autophagy might not be implicated or has a minor role in cancer-induced skeletal muscle wasting. As it is well known that the loss of autophagy leads to muscle wasting exacerbation in response to atrophic stimulus (Vainshtein and Sandri, 2020), further studies are needed to elucidate the variations of autophagic flux, whether it is an increase or a decrease, in cancer cachexia.

The FoxO family of transcription factors (FoxO1 and FoxO3 particularly) and NF-κB are known to be essential transcription factors implicated in the regulation of numerous genes of both UPS and autophagy pathways in various models of skeletal muscle atrophy (Vainshtein and Sandri, 2020). These transcription factors have been found to be upregulated in different cancer cachexia models (Cai et al., 2004; Lecker et al., 2004; White et al., 2011; Op den Kamp et al., 2013; Chacon-Cabrera et al., 2014, 2017; Gallot et al., 2014; Puppa et al., 2014; Chen M. C. et al., 2016; Sun et al., 2016; Sirago et al., 2017; Salazar-Degracia et al., 2018; Lee et al., 2019; Bae et al., 2020) including in cancer patients (Rhoads et al., 2010; Skorokhod et al., 2012; Puig-Vilanova et al., 2015; Johns et al., 2017).

Aside from studies presented in the section of this review dedicated to breast cancer clinical studies (Lønbro et al., 2017; Bohlen et al., 2018; Guigni et al., 2018; Mijwel et al., 2018b; Møller et al., 2019; Wilson et al., 2020), no preclinical study has been done in order to explore protein breakdown in breast cancer models. However, some pre-clinical studies explored the effect of doxorubicin on skeletal muscle and an increase in both UPS and autophagy pathways was suggested as MAFbx, beclin1, Atg12, Atg7, and LC3B-II/I ratio increased with doxorubicin treatment in mice and rats (Smuder et al., 2011; Kavazis et al., 2014; Hulmi et al., 2018; Montalvo et al., 2020). Importantly, only Montalvo et al. (2020) explored p62 protein levels and found no change in its expression, again emphasizing the need to obtain a more precise understanding of the autophagic pathway. Interestingly, Yu et al. (2014) also treated mice with doxorubicin and found no change in numerous autophagy markers. Altogether, pre-clinical studies demonstrated altered protein synthesis and breakdown mainly in response to doxorubicin administration in rodents, again emphasizing that these mechanisms may induce skeletal muscle wasting in breast cancer patients.

### Pro-inflammatory and TGF-β Family Cytokines

As a critical upstream of protein turnover alteration, inflammation plays a key role in the development of muscle wasting in cancer patients. Indeed, either released by the tumor or immune cells, pro-inflammatory cytokines like TNF-α, TWEAK, IL-6, IL-1β, IL-8, and INFγ have been found to be upregulated at a systemic level in animals (Costelli et al., 1993; Baltgalvis et al., 2008; Zhou et al., 2010; Toledo et al., 2016; Guo et al., 2017; Chen T. et al., 2018; Bae et al., 2020; Bernardo et al., 2020; Huot et al., 2020) and in cancer patients (Scott et al., 1996; DeJong et al., 2005; Moses et al., 2009; Skipworth et al., 2011; Op den Kamp et al., 2013; Puig-Vilanova et al., 2015; Johns et al., 2017; Riccardi et al., 2020). Importantly, from a study that included 661 breast cancer patients, systemic inflammatory cytokines were associated with a poor survival, reduced disease-specific survival and disease-free survival (Cho et al., 2018). These inflammatory cytokines have been also found to be upregulated within skeletal muscle in pre-clinical studies (Skipworth et al., 2011; Johnston et al., 2015; Chen M. C. et al., 2016; Hatakeyama et al., 2016; Chen L. et al., 2019; Lee et al., 2019; Bae et al., 2020), but none of these has been investigated in breast cancer models.

In addition to pro-inflammatory cytokines, two particular members of the TGF-β family have been particularly explored in cancer cachexia: myostatin (MSTN) and Activin A. MSTN clearly represents one of the most potent negative regulator of muscle growth and is known to act through its receptor ActRIIB and the subsequent activation of the SMAD2/SMAD3 cascade (Rodriguez et al., 2014). MSTN and/or its downstream targets have been found to be upregulated in many experiments on cancer cachexia (Costelli et al., 2008; Bonetto et al., 2009; Zhou et al., 2010; Murphy et al., 2011; Aversa et al., 2012; Padrão et al., 2013; Chacon-Cabrera et al., 2014; Silva et al., 2015; Chen M. C. et al., 2016; Sun et al., 2016; Chen M. C. et al., 2018; Salazar-Degracia et al., 2018; Lee et al., 2019; Huot et al., 2020), as well as in studies exploring the effect of doxorubicin administration (Kavazis et al., 2014; Liu et al., 2019). Acting through the same receptor than MSTN (ActRIIB), Activin A is also found to be increased in cancer cachexia (Leto et al., 2006; Loumaye et al., 2015; Matsuyama et al., 2015; Chen J. L. et al., 2016; Chen M. C. et al., 2016; Barreto et al., 2017; Zhong et al., 2019; Bernardo et al., 2020) and an independent prognosis factor of survival in cancer patients (Loumaye et al., 2017). Several authors conducted experiments with inhibition of the MSTN/Activin A pathway and found a reduction, or even a complete reversal, in the decrease of muscle mass and function in pre-clinical models (Liu et al., 2008; Benny Klimek et al., 2010; Murphy et al., 2011; Busquets et al., 2012a, b; Gallot et al., 2014; Hatakeyama et al., 2016; Levolger et al., 2019; Ojima et al., 2020; Pettersen et al., 2020), leading to the consideration of this pharmacological strategy for human cancer patients.

### Mitochondrial Alterations, Oxidative Stress, and Unfolded Protein Response

Mitochondrial alterations represent a major aspect of muscle deconditioning that have been already associated with skeletal muscle atrophy in breast cancer patients (Bohlen et al., 2018; Guigni et al., 2018; Mijwel et al., 2018b; Wilson et al., 2020), and in other cancers such as gastrointestinal and lung cancer patients (Op den Kamp et al., 2015; de Castro et al., 2019). Triggered by both structural and functional mitochondrial impairments, mitochondrial alterations have been particularly studied in pre-clinical studies. First, altered morphology and/or mitochondria loss have been found in different models of cancer in animals and/or with chemotherapeutic agents (Shum et al., 2012; White et al., 2012; Fontes-Oliveira et al., 2013; Barreto et al., 2016; Brown et al., 2017; Sorensen et al., 2017) as well as in gastric cancer patients (Zhang et al., 2020). Taken together, these results showing mitochondrial alterations on other cancer types strengthen the abovementioned results specifically observed in breast cancer (Guigni et al., 2018; Mijwel et al., 2018b) and might be a specific maladaptation between cancers. Concerning mitochondrial function, the overall oxidative pathway is clearly affected by both cancer and chemotherapeutic agents (Ushmorov et al., 1999; Constantinou et al., 2011; Julienne et al., 2012; Fermoselle et al., 2013; Gilliam et al., 2013, 2016; Padrão et al., 2013; Tzika et al., 2013; McLean et al., 2014; Gouspillou et al., 2015; Op den Kamp et al., 2015; Puig-Vilanova et al., 2015; de Lima Junior et al., 2016; Brown et al., 2017; Crouch et al., 2017; Pin et al., 2018; Ryan et al., 2018; Neyroud et al., 2019; Penna et al., 2019b; Hulmi et al., 2020; Kunzke et al., 2020). Among these studies, only two showed the potent negative impact of doxorubicin on complexes respiratory capacity (Gilliam et al., 2013; Gouspillou et al., 2015) while Crouch et al. (2017) highlighted a decrease in ATP production with cyclophosphamide administration, an immunosuppressor commonly associated with doxorubicin in breast cancer treatment. Interestingly, various authors also found altered mitochondrial dynamics, with a decreased fusion and increased fission, leading to mitochondria fragmentation in cancer cachexia (White et al., 2011, 2012; Barreto et al., 2016; Brown et al., 2017; Marzetti et al., 2017; Pin et al., 2018; de Castro et al., 2019; Huot et al., 2020). Surprisingly, although it was found that breast cancer patients lost mitochondria during their chemotherapeutic treatment (Guigni et al., 2018), mitochondria dynamics has not been investigated to date in specific preclinical models of breast cancer patients. Even if it is well known that mitochondria fission is prerequisite for the activation of the mitophagy process, it seems that mitophagy is also dysfunctional in cancer as several authors showed a decrease in key markers such as PINK1 or Parkin (Aversa et al., 2016; Marzetti et al., 2017). This statement has been also confirmed in the study of Gouspillou et al. (2015) with mice treated with doxorubicin (reduced Parkin protein levels) as well as in the study of Mijwel et al. (2018b) with breast cancer patients (reduced PINK1 protein levels).

As a consequence of mitochondrial dysfunction and potential reduced mitophagy, fragmented and damaged mitochondria accumulate in skeletal muscle and, in addition to being less bioenergetically efficient, produce excessive amounts of oxidative stress, mediated through increases in reactive oxygen species (ROS). Indeed, many different studies found an increase in ROS (Gilliam et al., 2013, 2016; Gouspillou et al., 2015; Min et al., 2015; Chacon-Cabrera et al., 2017; Pin et al., 2018; Ballarò et al., 2019; Montalvo et al., 2020), more specifically elevated levels of hydrogen peroxide (H_2_O_2_). Unanimously, several studies reported that doxorubicin administration in rodents led to an increase in H_2_O_2_ production (Gilliam et al., 2013, 2016; Min et al., 2015; Montalvo et al., 2020), while there is still no clinical study available to confirm this increase in breast cancer patients. One of the consequences of the increase in oxidative stress is the alteration of protein turnover pathways, with a decrease in protein synthesis, supported by an altered PI3k-Akt-mTOR pathway, and an increase in protein breakdown systems (i.e., UPS and autophagy). Aside the protein turnover deregulation, mitochondria-mediated oxidative stress is also a potent initiator of apoptosis [see reviews from Powers et al. (2016),Aggarwal et al. (2019), Sies and Jones (2020), and Hyatt and Powers (2021)]. Many studies showed an increase in key markers of apoptosis in various pre-clinical models (Belizário et al., 2001; Ishiko et al., 2001; Yoshida et al., 2001; Tsang et al., 2003; Figueras et al., 2004; Schwarzkopf et al., 2006; Baltgalvis et al., 2008; Murphy et al., 2011; Smuder et al., 2011; Chacon-Cabrera et al., 2014; Salazar-Degracia et al., 2016, 2018) and in cancer patients (Busquets et al., 2007; de Castro et al., 2019). Three other studies also explored the effect of doxorubicin treatment in rodents and *in vitro* (C2C12) and found increased levels of caspase 3 (both its activity and cleaved form of caspase 3 protein expression) and of Bax (Gilliam et al., 2012; Yu et al., 2014; Min et al., 2015). Finally, the study of Ahmadabadi et al. (2020) also observed a decrease in Bcl-2/Bax ratio in breast cancer-bearing mice, showing once again that apoptosis might be upregulated in breast cancer patients. Intuitively, the loss of muscle cells or myonuclei would appear like one of the causes of muscle atrophy, and studies have already shown associations between loss of muscle mass/CSA and the number of apoptotic cells (Allen et al., 1997; Borisov and Carlson, 2000; Smith et al., 2000; Dupont-Versteegden, 2005; Andrianjafiniony et al., 2010; Guo et al., 2012; Chacon-Cabrera et al., 2014; Cheema et al., 2015; Salazar-Degracia et al., 2016).

Increased levels in unfolded or misfolded proteins and oxidative stress (due to the potential deficit in autophagy/mitophagy and mitochondrial dysfunction) will lead to endoplasmic reticulum stress and trigger the unfolded protein response (UPR) that might represent another major maladaptation taking place during cancer cachexia. Acting through three pathways (PERK-eIf2α-ATF4, IRE1α-sXBP1, and ATF6-ATF6N) the UPR contributes to skeletal muscle atrophy by decreasing protein synthesis, increasing protein breakdown and, ultimately, inducing apoptosis (Urbina-Varela et al., 2020; Vainshtein and Sandri, 2020; Gallot and Bohnert, 2021). The UPR has been shown to be upregulated in several pre-clinical studies of cancer cachexia (Bohnert et al., 2016, 2019; Gallot et al., 2019; Straughn et al., 2021) and in response to doxorubicin treatment (Montalvo et al., 2020) leading to the conclusion that the increased activity of the UPR system would trigger the muscle atrophy program and contribute to muscle wasting. However, as clearly described in the review of Gallot and Bohnert (2021), specific increase in the PERK-eIf2α-ATF4 pathway might also be necessary during skeletal muscle atrophy to counteract it, as both pharmacological (Bohnert et al., 2016) or genetical tools (Gallot et al., 2019) aiming to inhibit this pathway aggravated cancer-related muscle atrophy. On the contrary, muscle-specific deletion of XBP1 in LLC-bearing mice exhibited a reduced muscle atrophy, demonstrating that the IRE1α-sXBP1 axis of the UPR system seems to be implicated in cancer-mediated muscle atrophy.

### Satellite Cells

The capacity of skeletal muscle to regenerate is another key parameter of its functionality. After injury, successful skeletal muscle regeneration appears to be driven by complex and precisely orchestrated processes involving multiple cell types. Of these cell types, satellite cells (SCs), localized between the sarcolemma and the basal lamina of myofibers (Mauro, 1961), represents the most studied and essential stem cells in order to support the regeneration process. In the context of cancer cachexia, several studies already showed that skeletal muscle tissue exhibited signs of ongoing degeneration/regeneration cycles, including ultrastructural damage, central nuclei localization, increased macrophages abundance as well as SCs proliferation in patients (Zampieri et al., 2010; He et al., 2013) and in pre-clinical models (Mehl et al., 2005; Chacon-Cabrera et al., 2014, 2017; Salazar-Degracia et al., 2016, 2018; Judge et al., 2018), including in breast cancer-bearing mice (Ahmadabadi et al., 2020). These signs of damage and regeneration might indicate an increased fragility of the skeletal muscle and an environment prone to lead to more degeneration/regeneration cycles. Having in mind that several authors also highlighted a clear decrease in regeneration capacity (He et al., 2013; Coletti et al., 2016; Inaba et al., 2018; Costamagna et al., 2020), cancer-related muscle atrophy may also result from muscle decreased repair/regrowth after injury and not only from different pathways causing protein turnover dysregulation. More specifically, the excellent study of He et al. (2013) demonstrated that SCs were able to proliferate and commit to the myogenic lineage, but unable to differentiate properly due to an NF-κB dependent increase in Pax7 expression. This increase in Pax7 expression was also found in breast cancer-bearing mice (Hesse et al., 2019) as well as in other cancers pre-clinical studies (Penna et al., 2010; Coletti et al., 2016; Costamagna et al., 2020), ultimately leading to muscle regeneration dysfunction. Importantly, D’Lugos et al. (2019) found that chronic doxorubicin administration drastically reduced SCs content in rats, suggesting that if cancer *per se* would inhibit myogenic differentiation process, the combination of both the disease and chemotherapeutic drugs administration might lead to global SCs dysfunction and loss in breast cancer patients. However, as highlighted in our section dedicated to clinical studies in breast cancer patients, only Mijwel et al. (2018b) investigated Pax7^+^-labeled SCs and found no change in their number. Therefore, more studies are necessary to clarify SCs fate and implication in breast cancer patients and/or pre-clinical models.

### Intermuscular Adipose Tissue and Fibro-Adipogenic Progenitors

The abnormal development of fibrotic and/or IMAT deposits within skeletal muscle is a strong marker of regenerative failure. As documented above, breast cancer patients exhibit an increase in IMAT (Rier et al., 2018; Reding et al., 2019; Beaudry et al., 2020), a result also found in other types of cancers [for a systematic review see Aleixo et al. (2020b)]. However, we did not find any study exploring the cellular mechanisms related to IMAT development in preclinical models of breast cancer or with the administration of commonly used chemotherapeutic agents. In muscle disuse or pathological conditions, such as Duchenne muscular dystrophy, FAPs proliferate and differentiate into adipose and/or fibrous tissue (Uezumi et al., 2011, 2014a; Ieronimakis et al., 2016) and are currently accepted to represent the major population that appears to play a role in IMAT development (Brioche et al., 2016; Biferali et al., 2019; Theret et al., 2021). In the context of cancer cachexia, one study found an increased presence of FAPs in the muscle environment of pancreatic cancer patients (Judge et al., 2018) that might explain the development of myosteatosis observed in overall cancer patients. Considering the increase in IMAT development found in breast cancer patients (Rier et al., 2018; Reding et al., 2019; Beaudry et al., 2020), it thus appears essential to explore FAPs fate in this specific context.

Other than their important role in muscle regeneration and abnormal development of IMAT, FAPs have been recently shown to promote skeletal muscle atrophy. Indeed, the study of Madaro et al. (2018) demonstrated that FAPs progressively accumulate and exhibit increased IL-6/STAT3 signaling, promoting muscle atrophy in different mouse models. Interestingly, inactivation of this pathway effectively countered the muscle atrophy and fibrosis observed in these models, emphasizing a potential role of FAPs secretome and paracrine effects on skeletal muscle fibers. Considering the ambivalent role of FAPs in the development of IMAT and muscle atrophy, further studies should focus on these stem cells in order to elucidate their potential role in both pre-clinical and clinical models of cancer-related skeletal muscle wasting.

## Conclusion

Breast cancer patients undergoing chemotherapy definitively experience skeletal muscle deconditioning, mainly characterized by both a decrease in muscle mass and function. Despite the fact that mechanisms of muscle deconditioning are well known in many other muscle wasting models, including in other pre-clinical or clinical models of cancers, they still remain relatively unknown in breast cancer patients. In fact, some studies using muscle biopsies highlighted protein turnover and mitochondrial alterations in breast cancer patients, but other studies are clearly needed to obtain a more precise understanding of the cellular processes implicated in breast cancer-mediated muscle deconditioning. This lack of knowledge inevitably leads to difficulties for the implementation of efficient preventive strategies such as exercise, nutrition or pharmacological treatments.

## Author Contributions

JM, EH, TH, and AP contributed to writing or editing the manuscript and approved the final version of the manuscript. All authors contributed to the article and approved the submitted version.

## Conflict of Interest

The authors declare that the research was conducted in the absence of any commercial or financial relationships that could be construed as a potential conflict of interest.

## Publisher’s Note

All claims expressed in this article are solely those of the authors and do not necessarily represent those of their affiliated organizations, or those of the publisher, the editors and the reviewers. Any product that may be evaluated in this article, or claim that may be made by its manufacturer, is not guaranteed or endorsed by the publisher.
